# Towards an evidence-based integrative lighting score: a proposed multi-level approach

**DOI:** 10.1080/07853890.2024.2381220

**Published:** 2024-07-25

**Authors:** Oliver Stefani, Isabel Schöllhorn, Mirjam Münch

**Affiliations:** a Lucerne School of Engineering and Architecture, Lucerne University of Applied Sciences and Arts, Horw, Switzerland; b Centre for Chronobiology, Psychiatric Hospital of the University of Basel, Basel, Switzerland; c Research Cluster Molecular Cognitive Neuroscience, University of Basel, Basel, Switzerland; d Department of Biomedicine, University of Basel, Basel, Switzerland

**Keywords:** Non-visual effects, daylight, lighting score, light quality, circadian, alertness, health

## Abstract

**Background:** Human circadian clocks are synchronized daily with the external light-dark cycle and entrained to the 24-hour day. There is increasing evidence that a lack of synchronization and circadian entrainment can lead to adverse health effects. Beyond vision, light plays a critical role in modulating many so-called non-visual functions, including sleep-wake cycles, alertness, mood and endocrine functions. To assess (and potentially optimize) the impact of light on non-visual functions, it is necessary to know the exact ‘dose’ (i.e. spectral irradiance and exposure duration at eye level) of 24-hour light exposures, but also to include metadata about the lighting environment, individual needs and resources.

**Problem statement:** To address this problem, a new assessment tool is needed that uses existing metrics to provide metadata and information about light quality and quantity from all sources. In this commentary, we discuss the need to develop an evidence-based integrative lighting score that is tailored to specific audiences and lighting environments. We will summarize the most compelling evidence from the literature and outline a future plan for developing such a lighting score using internationally accepted metrics, stakeholder and user feedback.

**Conclusion:** We propose a weighting system that combines light qualities with physiological and behavioral effects, and the use of mathematical modelling for an output score. Such a scoring system will facilitate a holistic assessment of a lighting environment, integrating all available light sources.

## KEY MESSAGES

- To assess the impact of light on non-visual functions, it is essential to know the exact ‘dose’ of 24-hour light exposures and metadata about the lighting environment and the individual.
- There is to date no measurement method available that allows a holistic assessment of a lighting environment for visual and non-visual aspects in humans.
- We discuss the need for an evidence-based integrative lighting score and outline a plan for its development.
- An evidence-based integrative lighting score, which includes a weighting system to combine light qualities with physiological and behavioral effects, will only be possible by joining forces, knowledge, and methodologies from many disciplines.
- Such a score entails the potential to sustainably assess, improve and maintain optimized lighting environments that promote the health and productivity of any cohort over the long term.

## 1. Introduction

Most physiological and behavioral functions in humans such as alertness, hormonal rhythms, metabolism, and sleep are modulated by circadian clocks across the 24-hour light-dark cycle [1,2]. Light-induced modulations of these functions are referred to as non-visual light effects, because they are transmitted via the eyes to the retino-hypothalamic tract to the core clock in the suprachiasmatic nuclei, and via various neuronal and hormonal pathways to the cells of the brain and the body. These effects depend mainly on a third class of photoreceptors in the eye, the intrinsically photosensitive retinal ganglion cells (ipRGCs) [3]. Timed retinal light exposure is therefore essential for synchronizing the circadian clocks to the external 24-hour solar day. Many laboratory and field studies have demonstrated the acute and circadian impact of light on physiological (e.g. sleep-wake timing, hormone secretion [4–7], metabolic functions [8,9]), and behavioral functions (alertness, mood, cognitive performance; [10–15]; for a review see [16]).

Not surprisingly, emerging evidence has shown that habitual light exposure impacts on health-related aspects in many ways – either directly, for example by changing circadian phases of sleep and wakefulness [17,18] - or on the long term, with higher risks of health deterioration, e.g. for psychiatric diseases with chronically higher light levels at night [19], or with higher risks for sleep disorders under chronically low light exposure levels during the day (e.g. [20]).

In addition to provide sufficient light for vision, innovative developments have been made in recent decades to consider also non-visual light effects at workplaces and in institutions through the implementation of new electric lighting systems. Most of these lighting systems (also known as dynamic, circadian, biodynamic or human-centric lighting), allow intensity and spectral composition to be adjusted across the 24-hour day [21,22]. For example, light-emitting diode (LED) lamps with so-called ‘tunable white lighting’ (containing two or more colors of LED chips) enable user-driven adjustments of illuminance and color temperature (i.e. warm, neutral or cold in appearance) based on the time of day, applications and occupant preference. This shift from improving only visual aspects of lighting towards optimizing also non-visual functions of light such as sleep (e.g. shorter time to fall asleep by reducing the short-wavelength proportion in the ambient lighting in the evening [23]), or increased alertness during the day in office workers (by enhancing ambient lighting with a greater proportion of the short-wavelength part of the spectrum [24]) has been timely and is ongoing.

This is also reflected in the recent developments of new lighting standards and recommendations for daytime, evening and nighttime light exposure. These recommendations are aimed to raise awareness towards tailored light and dark exposures in humans [25,26]. Recent recommendations by Brown et al. also include a method for estimating the dose and exposure profile to light [27], a system for dose and exposure assessment [28], and a proposal to unify multiple aspects of exposure [29].

Scheduled bright light exposure has long been known to improve depressive symptoms in patients [30–32]. Since its first use in the treatment of Seasonal Affective Disorders (SAD) more than 40 years ago [33], new evidence for the efficacy of light therapy in patients with psychiatric and other disorders has emerged [30,34–39]. These therapeutic effects have derived from commercially available high-intensity light treatment lamps [40]. More recently, ceiling-mounted dynamic and/or spectrally tunable electric lighting installations [41–44] were implemented to improve sleep, well-being and mood, for example in nursing homes [30,31,43,45,46]. Bright light therapy significantly reduced daytime sleepiness in patients with neurodegenerative disorders, i.e. Parkinson’s Disease [47]. Scheduled daylight exposure has also been shown to be an effective treatment for patients with SAD [48]. In addition, recent evidence suggests a causal relationship between less daylight per day and an increased risk of psychiatric diseases (e.g. depression) [49,50] or other diseases [20].

**Figure 1.**
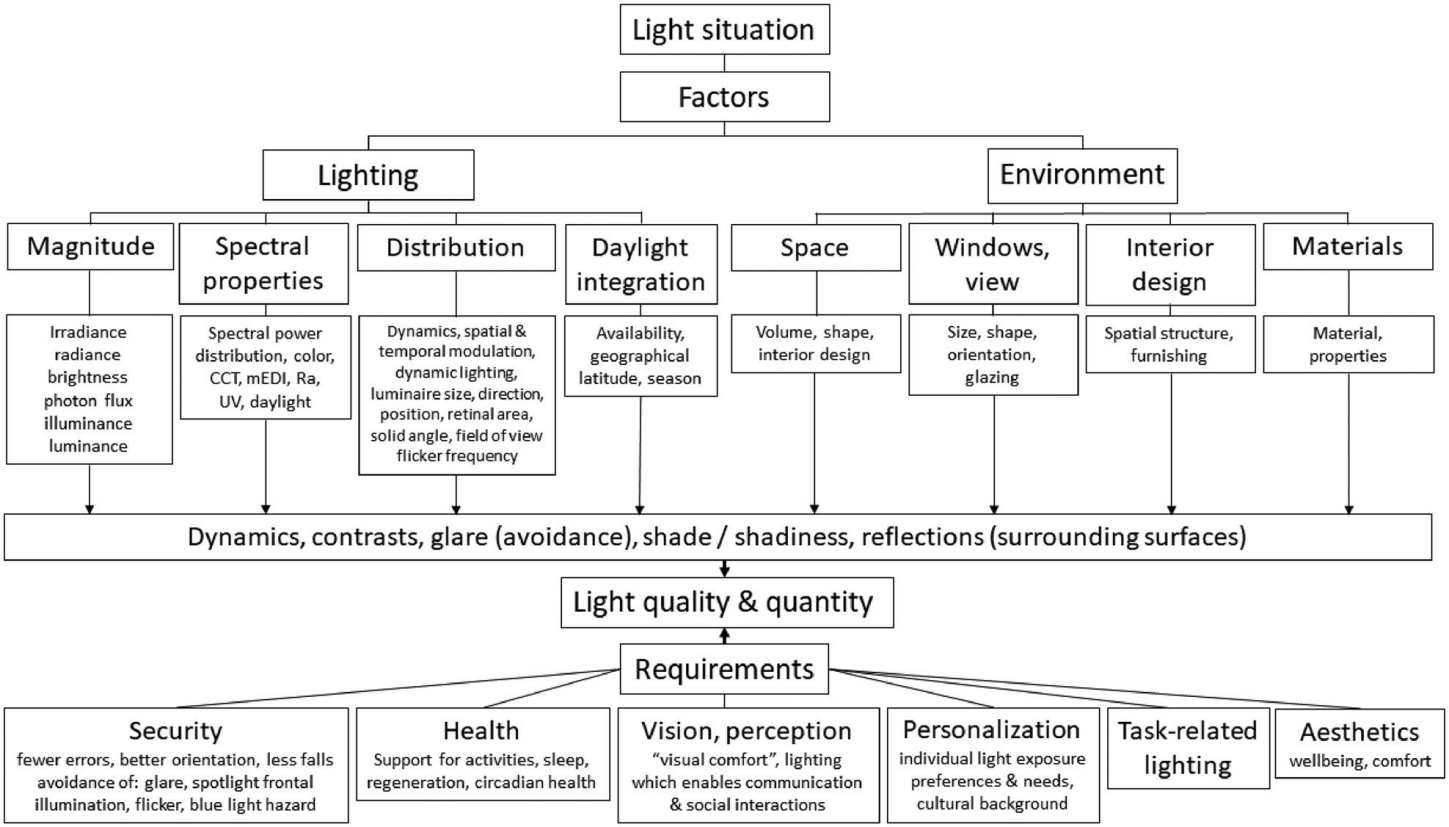
Schematic overview of the factors that determine light quality and quantity. The two main categories come either directly from lighting or via environmental factors. Both impact on light quality and quantity which in turn is defined by light requirements.

Despite these developments we cannot ignore the fact that many institutions, work places and homes do not provide the minimum recommended daytime light levels (i.e. 250 lx melanopic equivalent daylight illuminance; mEDI [25]) to support physiological and behavioural functions (e.g. alertness, well-being and cognitive performance) for most of the time during the day [51,52]. There are also many other gaps to be filled such as the importance of light quality and behavioral, cultural, social and intuitional influences of light. Figure 1 summarizes the most important known visual and non-visual light effects.

## 2. Problem statement

One of the unresolved problems in lighting research and light applications in human environments is that the required light ‘dose’ as an integrated function of exposure time, time of day, light qualities (i.e. spectral distribution, temporal and spatial dynamics) as well as the environment (i.e. room location, floor, geographical latitude), and individual behavior (e.g. based on differences in light sensitivity, the effects on mood and alertness) for an optimized light environment has not yet been elucidated. It is possible to assess each of these variables alone, but as stated above, there is no ‘all’ in one function predicting ideal light exposure.

Even if such a dose would be known, there is currently no method of comprehensively measuring and describing an individual’s light environment in terms of their visual and non-visual light-dependent potential (and other influencing factors). This would mean monitoring all the light a person receives through the eyes at any given time. To date, the only way to measure individual light exposure has been to use wearable light sensors [53–55], worn either near the eye [56], around the wrist or elsewhere [57] and to assess α-opic illuminances [58] or photopic illuminance. It is not yet possible, to derive predictions of long-term health and disease risks from these measures.

**Figure 2.**
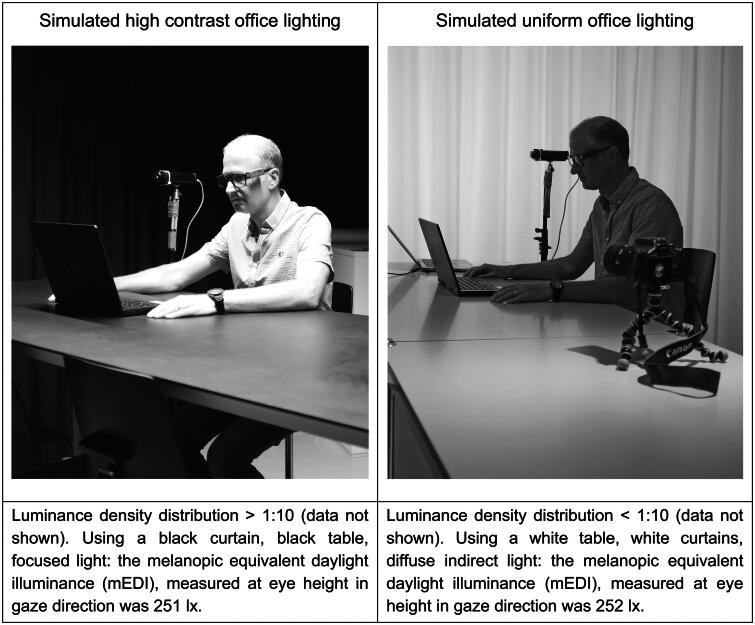
Illustration of two contrasting lighting designs with the same melanopic illuminance at eye level but different luminance distributions (photograph: Sina Plate) (consent to publish was provided as the shown person is co- authors of this paper).

Finally, measuring mEDI at eye level does not necessarily guarantee a comfortable lighting environment. As shown in Figure 2, if the contrast between field of view and ambient lighting is too high, this may induce discomfort glare.

Which brings us to the main point: effective methods and applications of holistic light (and dark) concepts, that integrate visual requirements, non-visual effects of light and lighting design aspects, have not yet been sufficiently developed and translated into a generally accepted and evidence-based framework. We have identified six key issues, that may prevent the development and application of better lighting scenarios in the field and in the clinic:

1. *Methodology:* To date, there is a lack of standardized and transferable procedures and measurement methods to derive quantification of non-visual light effects (such as alertness, cognitive performance, circadian entrainment, mood) in relation to indoor lighting environments and behavior in real world settings and for different individuals. Recently, there have been attempts to fill this gap for methods to assess light and to report non-visual functions of light in the laboratory [59,60]. To date, some dose-response curves for melanopic EDI or melanopic irradiance e.g.: for alertness [61] and melatonin suppression [29,62,63], sleep [63,64], pupil size and pupil light responses [65], as well as duration response curves for the pupil and the waking EEG [66] and other non-visual effects of light, allow for the estimation of these effects. The current recommendation for daytime light intensity in relation to alertness and melatonin suppression for a young and healthy person is 250 lux mEDI at the corneal plane in the vertical direction [25].
2. *Transferability:* Although numerous laboratory experiments have provided evidence that light fundamentally affects many psychological and physiological functions, it is difficult to translate this into everyday applications, because of the degree, to which these studies control the environment and the behavior of the participants. Many methodological differences in study design, choice of lighting conditions, assessment, geographical location and reporting of lighting and outcome variables as well as inter-individual differences [67] contribute to the heterogeneity even between laboratory studies. Although several reviews [29,62] have identified the mEDI as the best predictor for non-visual light effects on physiology and behaviour, all of the studies included in these reviews were conducted in the evening or at night and may not be generalizable to daytime. Few studies have measured the mEDI (or α-opic lx as in [68]) under more realistic conditions, including daylight (e.g. [69–72]).

1. *Applicability:* Existing recommendations for light levels related to non-visual effects of light lack specificity for different work and home environments, and for different target groups (e.g. schoolchildren, the elderly, or patients with major depression). Up to now, there are no common recommendations on how to further personalize light exposure and differences in light sensitivity.
2. *Independency:* In the last 20 years, many lighting companies have invested into research to develop new electric light sources and lighting systems. Various off-the-shelf systems have emerged. The difficulty is, that many of these systems are based on internally developed resources, that are not publicly available.
3. *Bias in terminology:* This has led to different reporting methods, that are not comparable between products. For example: many companies sell what they consider to be ‘healthy lighting’, although it is not understandable (and traceable) what the ‘health’ aspect is.
4. *Combination of electric light and daylight:* Many commercial lighting systems focus on electric light. As a result, there is little focus on improving workplace and building lighting to incorporate daylight or to encourage a combination of the two. A relatively small number of commercial approaches have been made to increase the availability of daylight in buildings or to provide automated control systems that allow the integration of electric light and daylight.

## 3. The idea of an evidence-based integrative lighting score (EBILS)

To address these issues (see above) - what could be a possible solution? We propose a comprehensive, holistic approach which would allow complex lighting situations to be assessed in terms of visual and more importantly, non-visual functions of light, rather than isolated effects from individual light sources. The idea is to develop a new evidence-based integrative lighting score (EBILS) that can be used in real-world settings and with different target groups. Such an approach would address the issues of methodology, transferability, applicability, and independence, bias in terminology and the combination of electric light and daylight (see above). In addition, a sustainable and widely accepted solution will require the active participation and multi-stage input of experts from diverse fields such as chronobiology, medicine, metrology, building physics, architecture, and lighting design.

In this commentary, we will further identify and summarize parameters of daytime electric lighting from existing meta-analyses, reviews and recommendations to support non-visual functions of light (summary of a narrative mini-review) and identify gaps in current knowledge. Secondly, we will propose the conceptual planned pathway for the development of an EBILS.

## 4. Evidence from the literature: a mini review

We conducted a search for relevant literature to identify the gaps in the knowledge about the effects of light on humans during the day. We focused on the results from existing reviews with or without meta-analyses, that already summarized the findings and/or made recommendations for daytime lighting (see Supplement for search criteria and Supplemental Figure S1 for a flow chart).

Although there are no specific laws for lighting projects that consider the effects of changing light on people, there are recommendations, such as technical reports from the CIE [22] (CIE S 026:2018 ‘CIE System for Metrology of Optical Radiation for ipRGC-Influenced Responses to Light’), from CIBSE [73] (Chartered Institution of Building Services Engineers) and from the Building Research establishment (BRE), the Research Insight Circadian lightingfrom UL (UL DG 24480 ‘Design Guideline for Promoting Circadian Entrainment with Light for Day-Active People’) and WELL [74] (The WELL standard ‘CIRCADIAN LIGHTING DESIGN’ and the update WELL v2 pilot), and nationally, there are other approaches e.g. the DIN from Germany: DGUV 215-210 ‘Non-visual Effects of Light on Humans’ (DIN SPEC 67600:2013-04 [75];technical report); and the ‘Biologically Effective Lighting - Planning Recommendations’ (DIN/TS 5031-100:2021-11 [76] ; Optical radiation physics and illuminating engineering - Part 100: Melanopic effects of ocular light on human beings - Quantities, symbols and action spectra).

Whilst evening and nighttime light effects would also require considerations for lighting recommendations for shift workers, we are concerned here with the development of the EBILS for daytime workers. Therefore, we will focus on daytime lighting, and we limited our literature search to daytime light effects. The main results of the 19 studies identified (daytime light exposure results only) are summarized in Table 1:

**Table 1.** Key findings from existing meta-analyses, literature reviews and recommendations for lighting and light-dependent non-visual functions.

Author [Reference]	Highlights of the key findings (with effects during the day only)
Cheshmeh Noor et al. [77] (*n* = 7)	Short-wavelength light during the early morning, significantly improved subjective and objective measures of alertness (derived from neural activity, assessed by the electroencephalogram; EEG). Red-appearing light (600 - 640 nm) during daytime can significantly improve subjective and objective measures of alertness.
Mu et al. [78] 1 (*n* = 19)	Light exposure shows significant improvement in subjective alertness [standardized mean difference (SMD) = −0.28, 95% confidence interval (CI): −0.49 to −0.06, *p* = 0.01] and objective alertness (SMD = −0.34, 95% CI: −0.68 to −0.01, *p* = 0.04). High correlated color temperature (CCT; i.e. > 5000 K) vs. low CCT (< 3000 K) improved subjective alertness (SMD = −0.37, 95% CI: –0.65 to –0.10 *p* = 0.007, I^2^ = 26%) and objective alertness (SMD = −0.36, 95% CI: −0.66 to −0.07, *p* = 0.02, I^2^ = 0).
Pachito et al. [79] 1 (*n* = 5)	High CCT vs. standard CCT potentially improves subjective alertness (SMD = −0.69, 95% CI: −1.28 to −0.10) in daytime workers. Little evidence for differences of better mood (MD = 2.08, 95% CI: −0.1 to 4.26), or worse mood (MD = −0.45, 95% CI: −1.84 to 0.94) between high CCT lighting and standard lighting.
Nixon et al. [80] 2 (*n* = 14)	None of the α-opic values (including mEDI) could be associated with a significant effect on mood. Longer exposures to light and exposures to light in the morning were associated with better mood.
Siraji et al. [42] 2 (*n* = 59)	Short-wavelength light exposure and higher intensity light exposures potentially improve subjective alertness (depending on time of day and homeostatic sleep drive). Not as straightforward as the acute alerting effect of light is the relationship between higher cognitive functions and light exposure. The optimal light intensity which can lead to better cognitive performance may depend on the light spectrum as well as on task complexity, and time of day.
Kompier et al. [81] 2 (*n* = 14)	Greater light exposure during the day may improve sleep during the subsequent night. Favorable impacts of dynamic lighting scenarios on human functioning are suggested, but established strategies for their description and assessment are presently missing.
Xu et al. [82] 2 (*n* = 11)	Short-wavelength light at low irradiance does not increase alertness during daytime, whereas polychromatic bright white light has the potential to reduce sleepiness during the day. Many environmental factors impact the magnitude of alerting effects of light during daytime: e.g. prior light history and inter-individual differences (including chronotype).
Souman et al. [83] 2 (*n* = 25)	Polychromatic white light at high photopic illuminance and high CCTs increases subjective alertness, likely due to activation of ipRGCs. Overall, it is difficult to draw definitive conclusions and more studies are needed to establish a dose-response curve of light and alertness during daytime.
Xiao et al. [84] 3 (*n* = 14)	Significant association between high photopic illuminance and higher subjective alertness during daytime. High photopic illuminance was associated with better mood in the morning, and worse mood in the afternoon.
Prayag et al. [85] 3 (*n* = 4)	Monochromatic blue light exposure during daytime leads to reduced objective sleepiness (assessed with electrophysiological markers). The right ‘dose’ of light during daytime can improve subsequent sleep quality.
Figueiro et al. [86] 3 (*n* = 15)	Light history is important and there is no time of day when there is no phase shifting effect from light exposure. Photopic illuminance greater than 2500 lux is increasing acute alertness (objectively and subjectively). Red light (peak wavelength at 630 nm) has alerting effects, too.
Lucas et al. [68] 3 and 4 (*n* = 5)	Maximum ipRGC activation can be achieved with high retinal irradiance and with a spectral composition towards the shorter wavelength range of light (i.e. green, blue). Predictions of non-visual effects from a given light source based on its intensity and spectral composition cannot yet be derived. Light measurements should quantify the effective irradiance weighted for each photoreceptor sensitivity (α-opic illuminance).
Lok et al. [87] 3 (*n* = 19)	Based on existing literature, the results of polychromatic white light effect on alertness are inconclusive, especially for objective alertness measures during daytime. Large differences between study designs make it difficult to compare results.
Stephenson et al. [88] 3 (*n* = 5)	Light therapy can positively influence mood in patients with mood disorders. The effects depend on light intensity, exposure duration and timing, as well as on the spectral composition of the light source. Feelings of vitality were improved after bright light exposure in office workers. Extended daylight exposure throughout the day has positive effects on circadian phase. Higher occurrence of sleep-disorders, fatigue, lack of concentration and worse mood in shift-workers working indoors.
Vandewalle et al. [14] 3 (-)	Cognitive brain responses are substantially modulated by light, as shown in fMRI studies. The magnitude of effects depends on a combination of light exposure duration, photon density and wavelengths. Exposure to blue-enriched white light during daytime increases subjective alertness and performance and reduces evening fatigue in office workers.
Cajochen [89] 3 (*n* = 4)	Light exposures substantially modulate non-visual effects of light for many biological functions, including subjective and objective alertness. Illuminance, duration, timing, and wavelength of light are most important for modulatory alerting effects.
Brown et al. [25] 4 (*n* = 5)	Expert consensus for healthy daytime and evening/night-time light environments with the following recommendations: Day: recommended minimum mEDI (melanopic equivalent daylight illuminance [58]) 250 lx vertically at the eye. Evening: reduced mEDI to 10 lx at least 3h before bedtime. Night: mEDI < 1 lx
Stefani et al. [26] 4 (-)	Evidence from current lighting standards and recommendations that address visual issues are currently not designed to address the impacts of non-visual light effects during the day. The proposed minimum mEDI at a vertical plane (i.e. > 250 lx mEDI [25]) is higher than that those recommended in current lighting design practices. The challenge will be to comply with existing guidelines to avoid discomfort glare [90,91] and achieve sufficiently high levels of mEDI at workplaces [25] during the day. This could be achieved by: Optimizing the spectral power distribution and vertical mEDI by implementing daylight availability and light sources with a relatively high mEDI during daytime. Importantly, this should be related to mEDI since correlated color temperature (CCT) is not an appropriate proxy for biological potential of light measures [92]. Optimizing lighting distribution (also considering the reflection of the surrounding surfaces).
Vetter et al. [93] 4 (-)	Exposure to bright days and dark nights is recommended. Natural daylight or white lighting with large proportions of short wavelengths during the day is beneficial even if exposure duration is short. Regular daily light exposure patterns.

Only results which included studies during the day were considered (for a list of search criteria, see Supplement). 1 = systematic review and meta-analysis; 2 = systematic review without meta-analysis; 3 = scoping or narrative review; 4 =recommendations, expert opinion; (in round brackets: the number of included studies conducted during the day).

The following summary condenses the frequency of reported effects from Table 1:

*Acute light effects on alertness were achieved with:*

- high intensity electric lighting in 42% of studies; i.e. 8 out of 19 studies, which was defined as: higher intensity (10.5%), bright white light (10.5%), and high illuminance (21%)
- red-looking lighting in 10.5% of studies (i.e. 2 out of 19)
- light with a greater proportion of the shorter wavelength spectrum of visible light, mentioned in 42% of studies (i.e. 8 out of 19 studies), when summarizing: enriched in short-wavelength lighting (16%), high correlated color temperature (CCT) (16%), monochromatic ‘blue’ (5%), blue-enriched (5%)
- longer duration of light exposure (5%)

*Positive light effects on mood were found with:*

- longer exposure durations (10.5%; i.e. in 2 out of 19 studies)
- light exposure in the morning (16%; i.e. 3 out of 19 studies)
- higher CCT (10.5%; i.e. 2 out of 19 studies)
- higher photopic illuminance (10.5%; i.e. 2 out of 19 studies)

*Cognitive brain responses were modulated by light, the effects depended on:*

- exposure duration (5%; i.e. 1 out of 19 studies)
- photon density (5%; i.e. 1 out of 19 studies)
- wavelength (5%; i.e. 1 out of 19 studies)

Based on the key findings above summarized, we conclude that different quantities and qualities of lighting have the potential to increase objective and subjective alertness, reduce sleepiness, increase activity, modulate brain responses, improve mood and increase vitality and possibly improve cognition during the day and sleep at night. There are some recommendations for minimum daytime lighting available (≥ 250 lx mEDI; in a vertical direction at the eye level [25]). These recommendations derive from laboratory studies where alertness and melatonin suppression were carefully monitored in selected study participants during the evening and at night. However, the optimal ‘light dose’ - a combination of light intensity, spectral composition and exposure duration - needed to achieve tailored effects for different non-visual (and visual) effects during the day for an individual, cannot yet be determined.

One limitation of this mini review is that it only considered reviews (with studies already included), so it may not be conclusive, and we certainly missed also some other reviews. A large systematic meta-analysis is needed to include and report the weighted effects of as many studies as possible that have investigated the effects of light on humans during the day. Another limitation is that there were some conflicting results, for example, that red light has an alerting effect, too [77,86,94]. Recently it has been shown in humans, that the color of a light source per se has no effect on non-visual functions [95]. These effects are mainly driven by the melanopic EDI of a light source. In the future, more carefully conducted field and laboratory studies are needed to pave the way towards detailed and even personalized recommendations.

## 5. Lighting parameters to be addressed for the establishment of an EBILS

The list of variables to be considered in the development of EBILS is rather long, and includes variables related to 1) visual functions (e.g. glare, luminance ratios, flicker, color rendering), see e.g. references [90,96,97], 2) to non-visual functions of light (e.g. magnitude, spectral distribution, timing (see Table 1), and could 3) include new metrics such as the Daylight Spectrum Index (DSI) [98]. As the EBILS will deal with lighting during the day, it will be important to take this into account:

### 5.1. Daylight availability

Compliance with standards such as EN 17037 (Daylighting in Buildings) and other daylighting quality metrics, e.g. Spatial Daylight Autonomy (sDA) and Annual Sunlight Exposure (ASE) [99] need to be confirmed. In working environments without access to daylight, the possibility of spending time outdoors during breaks must be provided. In addition, the spectrum of electric light should be close to that of daylight. Assuming a person sleeps for 8 h and has 3 h before bedtime below the recommended 10 lx mEDI, the total daytime light ‘dose’ would be 13 h at 250 lx mEDI, resulting in a minimum recommended daily light exposure of 3,250 lux hours (lx*h). In a study by Adamsson et al. (2017) [100] light exposure was approximately 23,000 lx*h in summer and 1,500 lx*h in winter. This exposure is 0.5 to 7 times higher than the minimum recommendations for indoor lighting.

### 5.2. Consideration of seasonal differences

Changes in seasonal daylength and solar irradiance drive long-term adaptations to retinal light sensitivity as it was shown for the pupillary light reflex to light, melatonin suppression and sleep wake timing [101–105]. Sensitivity to light in the evening (as indexed by melatonin suppression) is indeed reduced in summer, probably due to higher daytime light exposure [102,106–108]. In the study by Schöllhorn et al. (2023), in summer, the evening light exposure in the experimental light condition had no effect on sleep onset time when exposed to a high melanopic display light. In winter however, sleep latency was significantly prolonged by the evening light exposure. Short-term light history also influences human responses to light, for example in the evening. This was shown for melatonin suppression to evening light exposure, alertness and cognitive performance [106,108–110].

### 5.3. Assessment of contrasts and optimized light distribution

An optimized light distribution by minimizing discomfort glare can be achieved by lowering blinds or closing curtains. The use of existing automated glare assessments (e.g. Daylight Glare Probability Index (DGP) [111]) may be applied to develop automated lighting regulation systems in the future. To illustrate that with the same mEDI at eye level many different scenarios are possible, Figure 2 illustrates two different lighting settings in an experimental test bed (HSLU Lucerne, Switzerland). The left image shows an office with 251 lx mEDI at eye level, but with high contrast, which might cause discomfort glare. The image on the right shows an office with 252 lx mEDI at eye level and with uniform light distribution. Although the overall lighting on the right side appears darker than on the left side, the user shown on the photograph (OS) receives the same light dose at eye level.

Most of the existing studies make no or marginally allowance for head position, angle of gaze, and retinal irradiance. All these factors contribute to the amount of light that can reach the retina. Therefore, the following factors should also be considered:

### 5.4. Angle of gaze

Depending on the spatial arrangement of a lighting situation, the real-time exposure to light reaching a person’s eye is a complex process since it involves head and eye movements. Therefore, a single light measurement at a defined location does not account for these temporal and spatial variations in light exposure at the human eye.

### 5.5. Retinal irradiance

- Pupil size: One of the limiting factors for retinal irradiance is pupil size. Therefore, an estimate of the luminous flux received by the retina through the variable pupil size from a luminous environment needs to be taken into account [112]. At low illuminances, it has been recently shown, that the pupil size decreases in a dose-dependent manner with increasing mEDI (between ∼5 and 150 mEDI) [65], and therefore, corneal illuminance, measured with an illuminance meter does not precisely estimate the amount of light received at the retina (and the ipRGCs). Furthermore, lens transmittance and pupil size depends on the age and hence, non-visual effects such as melatonin suppression can change with age [113].
- Visual field: The second limiting factor of retinal irradiance is the dynamic visual field of the human eye and of course interactions with changes in pupil size. Ideally, this would include a precise definition of the binocular visual field of the human eye. In particular, visual fields are also dependent on ambient light levels as the eyelid provides a greater shielding angle to the eyes in bright environments.

In addition, other factors such as human eye convergence, disparity and facial shield angles (cheeks, nose, eyelids, etc.) should be addressed. While most illuminance meters account for light coming from a hemisphere covering a 180° field of view, instead, the additional use of a ‘field of view occlusion device’ for measurements at eye level has recently been promoted [114]. For field measurements, it could be considered whether the real-time gaze points can be recorded with an eye tracker. It should be noted that the calculation of the pupil apparent area is based on photopic vision, and may be different for the non-visual (melanopic) effect of light on pupil size also because of the distinct photoreceptor distribution on the retina.

Finally, the EBILS should be adaptable to incorporate new knowledge and to include other lighting parameters, and modules, e.g. for energy consumption, temperature regulation; or new knowledge about the contribution of single photoreceptors to specific non-visual effects of light as a function of exposure duration or age. Extension to other lighting parameters should include a reliable measure of inter-individual light sensitivity and it could also be adaptable to future extensions to evening and nighttime light exposure for example for shift workers.

## 6. Proposed pathway for the development of the EBILS

Here we will first describe the objectives and the concept of the EBILS (6.1), the proposed approach for its development (6.2), and the description of its concrete potential use in the field (6.3).

### 6.1. General concept

Our aim is to develop an evidence-based integrative lighting score (EBILS) which is a data driven approach: it will be sensitive, reliable, informative, easy to use, low cost and tailored to specific target groups and lighting environments, while using internationally accepted metrics. Such a tool will ultimately be an evidence-based integrated method for assessing and reporting lighting quality of a given indoor environment during the day (and in the future at night). The new approach is to use the EBILS not for individual light sources but to evaluate an entire lighting situation by weighting different metrics and metadata in relation to potential visual and non-visual functions of light and expressing them synergistically in a score, similar to what exists for example on labels for energy saving devices [115]. The EBILS is intended to be an open source, non-profit tool.

The development of the EBILS will require a weighting of factors, several validation steps and will need mathematical modelling process for reliability, practicality, and quality measures. The final tool will be a validated and accepted rating system comparable to already existing evaluation systems e.g. for green buildings (the international LEED rating system [116] and the Swiss SNBS rating system [117]). Such a tool will be based on existing scientific evidence, and will be reliable, informative, transparent and sustainable and following the FAIR principles (Findable, Accessible, Interoperable, and Reusable) [118]. Ultimately, it will contribute to and inform decision-making processes for revisions of current indoor lighting regulations for human occupants.

### 6.2. Proposed approach to develop the EBILS

**Figure 3.**
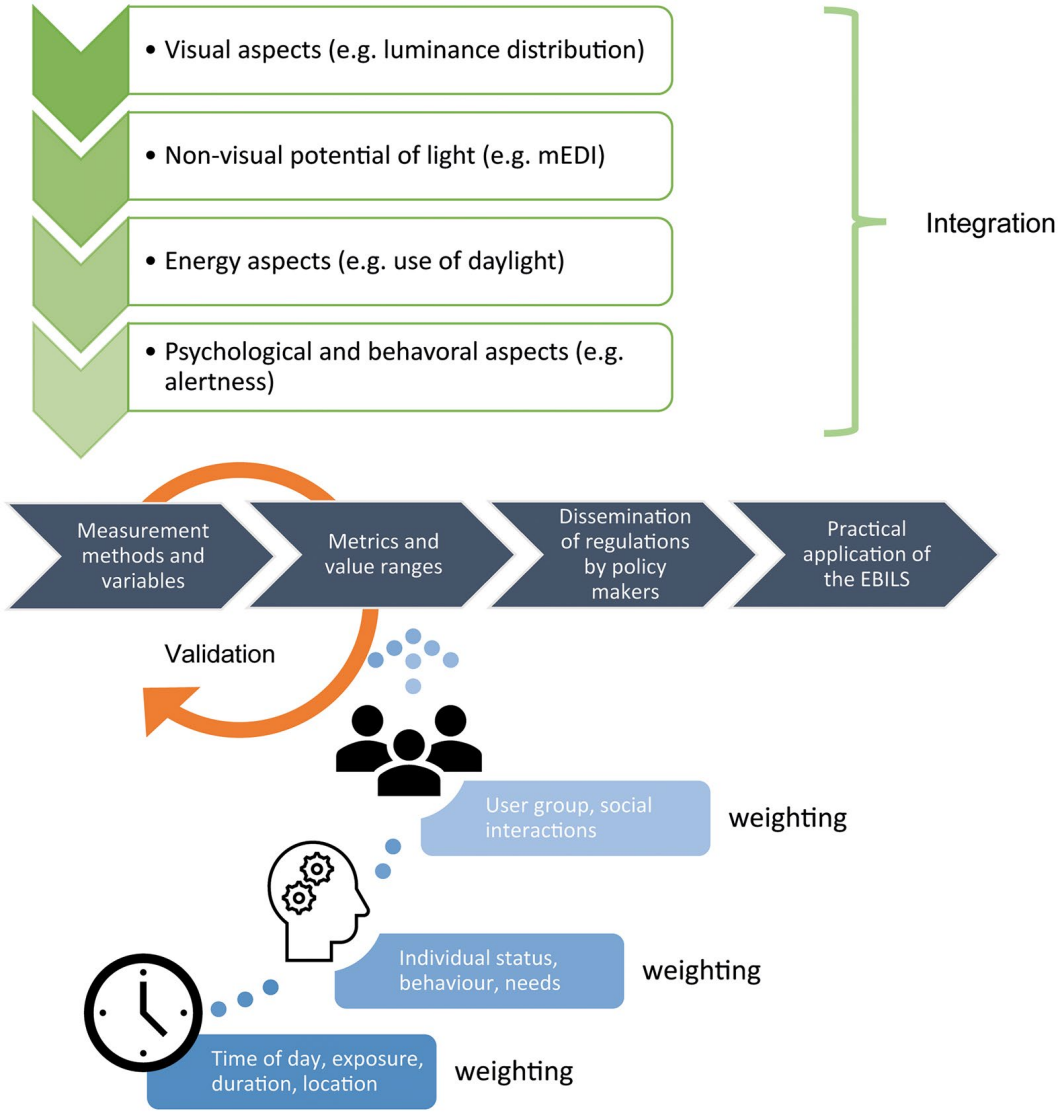
Schematic overview of the EBILS development and validation process: 1) integrated measurement categories (green arrows at the top) and, 2) metadata information (blue rectangles) form: the decision-making process to determine: 3) metrics and value ranges of lighting parameters (indicated by the first two horizontal dark grey arrows). The four horizontal dark grey arrows indicate the main pathway, with the last two rectangles forming rather a long-term process. 4) the orange arrow indicates the multilevel validation processes to establish an EBILS. Please note, the consultation and decision, making process with stakeholders occurs at each of the four main process steps, indicated by the horizontal dark grey arrows.

From existing empirical models (e.g. dose-response curves, available data sets, literature); standards; opinions and input from stakeholders and experts in the field, the following steps will be necessary:

- ***Identification*** of the most important lighting metrics to assess a lit environment in terms of visual and non-visual functions of light. We will begin with a larger model and aim to reach a parsimony model (3-4 lighting parameters, for example: vertical irradiance, mEDI, contrast). This will depend on the accuracy of prediction of outcomes variables (see below). There will be a co-design process with stakeholders to identify these metrics.
- ***Definition*** of the key behavioural outcomes for a given exemplary lighting setting (Figure 3, green vertical arrows). The defined behavioural variables could be e.g. subjective alertness, mood, and well-being, but this will be based on existing evidence from studies, stakeholder and user needs.
- ***Creation*** of an evidence-based weighted integration based on the variables defined above (Figure 3, blue dotted lines). It assigns a lighting metric to a psychological or behavioral output (see above). This is an iterative process and also involves existing mathematical models such deep learning tools to refine and adjust the function.
- ***Validation*** of a given weighting function: This is one of the most critical steps and is carried out as an iterative process involving stakeholder input at various stages and testing with real human occupants. Data from existing databases may also be used and mathematical tools such as neuronal network tools and existing model predictions [119–122] should assist the process of finding the best weighting function to match the lighting environment to a physiological or behavioural output. The aim is to achieve high sensitivity and reliability for different dynamic lighting situations. An EBILS should be iteratively validated in different exemplary lighting scenarios and used to predict new scenes. The iterative refinement should involve several workshops with experts in the field (from medicine, biology, lighting design, engineering, standardization, metrology etc.) who will critically review the prototype EBILS (Delphi process).
- ***Mathematical modelling*** tools (e.g. machine learning tools such as the random forest and Bayesian networks) will be used to incorporate metadata, stakeholder and user input and produce a final score. The goal is to achieve a high number of theoretical scores that can be assigned to different combined light/behavioural outcomes. These scores will be validated in test-beds i.e. in a predefined and standardized room where luminaires and furniture need to be set up in a standardized way and multiple light measurements need to be taken at defined positions in that room. This will form the prototype EBILS (Figure 3; first two horizontal grey arrows on the left), which will then be tested in real field situations (Figure 3, orange arrow). Adjustments will be made as necessary.
- ***Consensus and dissemination:*** The production of a working document should be circulated among a group of interested institutions to obtain further input from professional stakeholders. Once such a project is funded a consensus document with standardized operating procedures for the EBILS will be produced. The long term aim of the EBILS is to be endorsed by national (and international) stakeholders and policy makers, and to be incorporated into formal policy tools and recommendations. An additional workshop with stakeholders will be held and new published literature evidence will be added.

There will be, of course several risks to the final development. A major risk will be finding an appropriate weighting function, as there may not be enough scientific evidence for certain psychological and behavioral output variables with given lighting metrics and available metadata for user needs.

### 6.3. The assessment of a lighting situation with an EBILS

**Figure 4.**
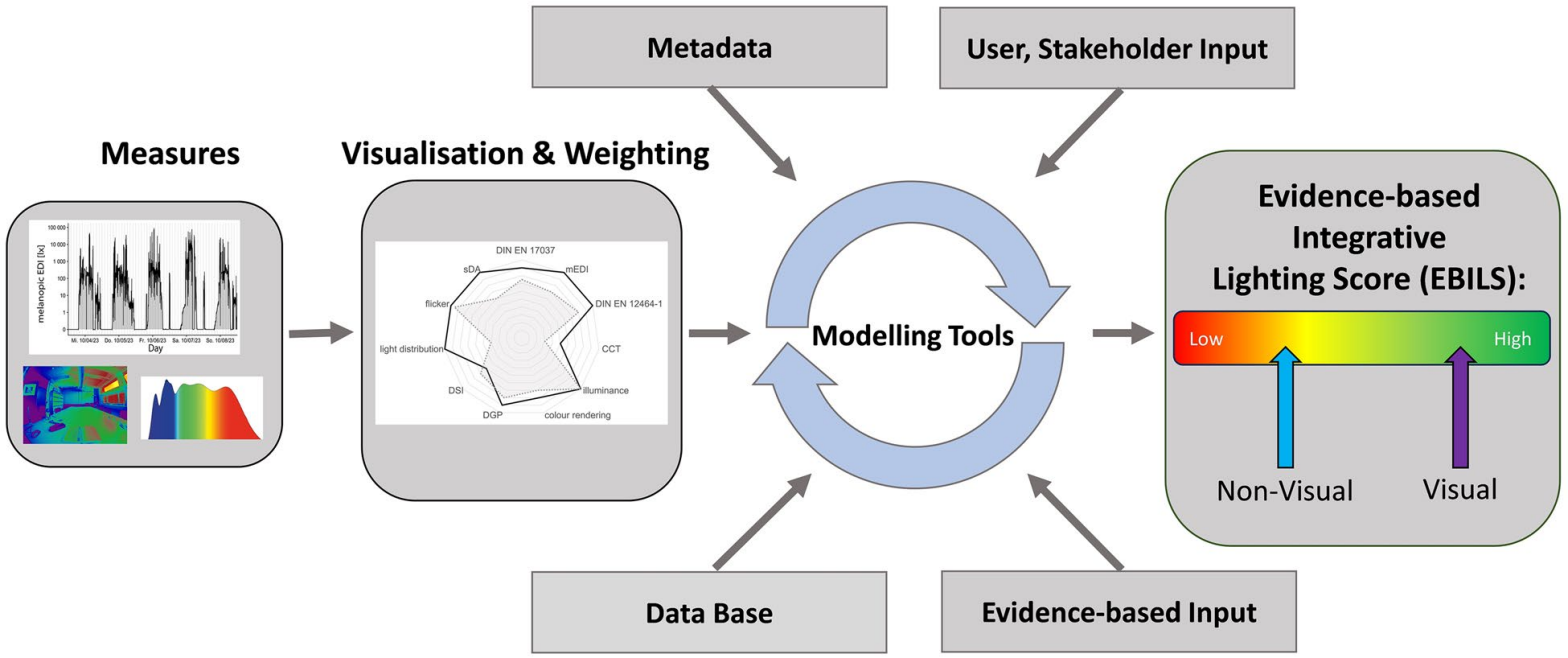
Conceptual overview for the planned generation of an EBILS output from a given lighting scene. 1) Physical assessment from a scene 2) weighted and visualized (shown as spider diagram): visual and non-visual lighting categories are measured and normalized (e.g. mEDI, light distribution, CCT, EN 17037), indicated by the dotted line. They all have various nominal values for visual and non-visual light-dependent functions, i.e. ‘optimal values’, derived from existing evidence (indicated by the grey solid line). 3) This data enters together with case specific available metadata (e.g. age, time of day, season, work requirements) and user feedback. Also, stakeholder input, validation data from data bases and new evidence-based studies will be regularly fed to the mathematical modelling tools. These will use machine learning tools e.g. random forest and Bayesian networks. 4) The output will be a predictive final EBILS output score ranging from low to high, indicated by the red (left) to green (right) colored range. It will be possible, to create an EBILS for visual (violet arrow) and non-visual scores (blue arrow) separately.

Once the final EBILS approach has been developed and can be assessed via a GUI (graphical user interface) that is user-friendly and app-based, the following steps will be necessary to assess a real lighting scene:

- Measure: The EBILS will be assessed in the field according to a predefined and standardized protocol where multiple light measurements have to be taken at defined positions (e.g. sitting in front of a computer screen, measuring in the direction of view of all computer workstations in the room), using different devices. It is also conceivable that measurements will be taken by a trained specialist wearing a mini-spectrometer or a similar device near the eye and recording continuous measurements following a standardized procedure (e.g. following predefined paths through a room using augmented reality). The next steps are performed offline:
- Weighting and Visualization: (Figure 4 below): Based on the validated weighting tools (see 6.2), each of the environmental light variables can be associated with a certain behavioral output and visualized e.g. with the spider graph.
- Add-ons: Available metadata information, user and stakeholder input will also be considered for the mathematical modelling (see below). Data from existing data bases could also be incorporated into the modelling tools, see below.
- Mathematical modelling tools: machine learning tools (e.g. random forest and or Bayesian networks) will produce a final EBILS for visual and light-dependent non-visual variables and the given lighting environment. The final score is a number and can be separated into high and low visual and non-visual aspects of light.

## 7. Limitations and conclusions

The limitations of this commentary are that we have not discussed visual aspects and overall lighting quality in detail as this would have been beyond the scope of this article. In our future work, as we develop the EBILS we will need to carefully consider also the visual aspects. Another limitation is that the EBILS will be a static assessment and will need to be repeated (or simulated) for different weather, seasonal and user specific conditions as well as prior light history. A major limitation is that it is not yet known how to better tailor light exposure to individual needs and how to cope with inter-individual differences.

To conclude, the proposed development of an EBILS is ambitious and will only be possible through the synergistically joining of forces, knowledge and methodologies from many disciplines. A final EBILS could be widely used in practice. It entails the potential to sustainably assess, improve and maintain optimized lighting environments that promote the health and productivity in any cohorts over the long-term.

## Supplementary Material

Commentary_Supplement_EBLS_Stefani_et_al_final_V2.docx

## Data Availability

All authors agree to make data and materials supporting the results or analyses presented in their paper available upon reasonable request. This commentary contains no original data, the search criteria of the scoping mini-review are listed in the supplement.

## References

[1] Aschoff J. Circadian rhythms in man. Science. 1965;148(3676):1427–1432. doi:10.1126/science.148.3676.1427. 14294139

[2] Czeisler CA, Gooley JJ. Sleep and circadian rhythms in humans. Cold Spring Harb Symp Quant Biol. 2007;72(1):579–597. doi:10.1101/sqb.2007.72.064. 18419318

[3] Berson DM, Dunn FA, Takao M. Phototransduction by retinal ganglion cells that set the circadian clock. Science. 2002;295(5557):1070–1073. doi:10.1126/science.1067262. 11834835

[4] Boivin DB, Duffy JF, Kronauer RE, et al. Dose-response relationships for resetting of human circadian clock by light. Nature. 1996;379(6565):540–542. doi:10.1038/379540a0. 8596632

[5] Khalsa SBS, Jewett ME, Cajochen C, et al. A phase response curve to single bright light pulses in human subjects. J Physiol. 2003;549(Pt 3):945–952. doi:10.1113/jphysiol.2003.040477. 12717008 PMC2342968

[6] Lewy AJ. Effects of light on human melatonin production and the human circadian system. Prog Neuropsychopharmacol Biol Psychiatry. 1983;7(4-6):551–556. doi:10.1016/0278-5846(83)90024-6. 6686693

[7] St Hilaire MA, Ámundadóttir ML, Rahman SA, et al. The spectral sensitivity of human circadian phase resetting and melatonin suppression to light changes dynamically with light duration. Proc Natl Acad Sci U S A. 2022;119(51):e2205301119. doi:10.1073/pnas.2205301119. 36508661 PMC9907124

[8] Gubin D, Danilenko K, Stefani O, et al. Blue light and temperature actigraphy measures predicting metabolic health are linked to melatonin receptor polymorphism. Biology. 2023;13(1):22. doi:10.3390/biology13010022. 38248453 PMC10813279

[9] Ishihara A, Courville AB, Chen KY. The Complex Effects of Light on Metabolism in Humans. Nutrients. 2023;15(6):1391. doi:10.3390/nu15061391. 36986120 PMC10056135

[10] Cajochen C, Zeitzer JM, Czeisler CA, et al. Dose-response relationship for light intensity and ocular and electroencephalographic correlates of human alertness. Behav Brain Res. 2000;115(1):75–83. doi:10.1016/s0166-4328(00)00236-9. 10996410

[11] Chellappa SL, Steiner R, Blattner P, et al. Non-visual effects of light on melatonin, alertness and cognitive performance: can blue-enriched light keep us alert? PloS One. 2011;6(1):e16429. doi:10.1371/journal.pone.0016429. 21298068 PMC3027693

[12] Milosavljevic N. How does light regulate mood and behavioral state? Clocks Sleep. 2019;1(3):319–331. doi:10.3390/clockssleep1030027. 33089172 PMC7445808

[13] Rahman SA, Flynn-Evans EE, Aeschbach D, et al. Diurnal spectral sensitivity of the acute alerting effects of light. Sleep. 2014;37(2):271–281. doi:10.5665/sleep.3396. 24501435 PMC3900613

[14] Vandewalle G, Maquet P, Dijk D-J. Light as a modulator of cognitive brain function. Trends Cogn Sci. 2009;13(10):429–438. doi:10.1016/j.tics.2009.07.004. 19748817

[15] Vandewalle G, Schwartz S, Grandjean D, et al. Spectral quality of light modulates emotional brain responses in humans. Proc Natl Acad Sci U S A. 2010;107(45):19549–19554. doi:10.1073/pnas.1010180107. 20974959 PMC2984196

[16] Dijk D-J, Archer SN. Light, sleep, and circadian rhythms: together again. PLoS Biol. 2009;7(6):e1000145. doi:10.1371/journal.pbio.1000145. 19547745 PMC2691600

[17] Ricketts EJ, Joyce DS, Rissman AJ, et al. Electric lighting, adolescent sleep and circadian outcomes, and recommendations for improving light health. Sleep Med Rev. 2022;64:101667. doi:10.1016/j.smrv.2022.101667. 36064209 PMC10693907

[18] Touitou Y. The double face of light effects: circadian adjustment or disruption. J Basic Clin Physiol Pharmacol. 2017;28(4):293–294. doi:10.1515/jbcpp-2017-0093. 28665802

[19] Burns A, Windred D, Rutter M, et al. Low daytime light and bright night-time light are associated with psychiatric disorders: an objective light study in >85,000 UK Biobank participants. medRxiv. 2022.

[20] Leger D, Bayon V, Elbaz M, et al. Underexposure to light at work and its association to insomnia and sleepiness: a cross-sectional study of 13,296 workers of one transportation company. J Psychosom Res. 2011;70(1):29–36. doi:10.1016/j.jpsychores.2010.09.006. 21193098

[21] CIE JTC 8. CIE S 017/E:2020 ILV: international lighting vocabulary. 2nd ed. International Commission on Illumination (CIE).

[22] Schlangen LJM. CIE position statement on non-visual effects of light. Recommending Proper Light at the Proper Time. 2019.

[23] Stefani O, Freyburger M, Veitz S, et al. Changing color and intensity of LED lighting across the day impacts on circadian melatonin rhythms and sleep in healthy men. J Pineal Res. 2021;70(3):e12714. doi:10.1111/jpi.12714. 33378563

[24] Viola AU, James LM, Schlangen LJM, et al. Blue-enriched white light in the workplace improves self-reported alertness, performance and sleep quality. Scand J Work Environ Health. 2008;34(4):297–306. doi:10.5271/sjweh.1268. 18815716

[25] Brown TM, Brainard GC, Cajochen C, Jr, and, et al. Recommendations for daytime, evening, and nighttime indoor light exposure to best support physiology, sleep, and wakefulness in healthy adults. PLoS Biol. 2022;20(3):e3001571. doi:10.1371/journal.pbio.3001571. 35298459 PMC8929548

[26] Stefani O, Cajochen C. Should we re-think regulations and standards for lighting at workplaces? a practice review on existing lighting recommendations. Front Psychiatry. 2021;12:652161. doi:10.3389/fpsyt.2021.652161. 34054611 PMC8155670

[27] Price LLA. On the role of exponential smoothing in circadian dosimetry. Photochem Photobiol. 2014;90(5):1184–1192. doi:10.1111/php.12282. 24749696

[28] Rea MS, Figueiro MG. Light as a circadian stimulus for architectural lighting. Light Res Technol. 2016;50(4):497–510. doi:10.1177/1477153516682368.

[29] Giménez MC, Stefani O, Cajochen C, et al. Predicting melatonin suppression by light in humans: unifying photoreceptor-based equivalent daylight illuminances, spectral composition, timing and duration of light exposure. J Pineal Res. 2022;72(2):e12786. doi:10.1111/jpi.12786. 34981572 PMC9285453

[30] Penders TM, Stanciu CN, Schoemann AM, et al. Bright light therapy as augmentation of pharmacotherapy for treatment of depression: a systematic review and meta-analysis. Prim Care Companion CNS Disord. 2016;18(5). doi:10.4088/PCC.15r01906. 27835725

[31] Tao L, Jiang R, Zhang K, et al. Light therapy in non-seasonal depression: an update meta-analysis. Psychiatry Res. 2020;291:113247. doi:10.1016/j.psychres.2020.113247. 32622169

[32] Zhao X, Ma J, Wu S, et al. Light therapy for older patients with non-seasonal depression: A systematic review and meta-analysis. J Affect Disord. 2018;232:291–299. doi:10.1016/j.jad.2018.02.041. 29500957

[33] Rosenthal NE, Sack DA, Skwerer RG, et al. Phototherapy for seasonal affective disorder. J Biol Rhythms. 1988;3(2):101–120. doi:10.1177/074873048800300202. 2979634

[34] Blume C, Garbazza C, Spitschan M. Effects of light on human circadian rhythms, sleep and mood. Somnologie (Berl). 2019;23(3):147–156. doi:10.1007/s11818-019-00215-x. 31534436 PMC6751071

[35] Dallaspezia S, Benedetti F. Antidepressant light therapy for bipolar patients: a meta-analyses. J Affect Disord. 2020;274:943–948. doi:10.1016/j.jad.2020.05.104. 32664036

[36] Dong C, Shi H, Liu P, et al. A critical overview of systematic reviews and meta-analyses of light therapy for non-seasonal depression. Psychiatry Res. 2022;314:114686. doi:10.1016/j.psychres.2022.114686. 35753223

[37] Garbazza C, Cirignotta F, D’Agostino A, et al. Sustained remission from perinatal depression after bright light therapy: a pilot randomised, placebo-controlled trial. Acta Psychiatr Scand. 2022;146(4):350–356. doi:10.1111/acps.13482. 35876837 PMC9804451

[38] Hirakawa H, Terao T, Muronaga M, et al. Adjunctive bright light therapy for treating bipolar depression: a systematic review and meta-analysis of randomized controlled trials. Brain Behav. 2020;10(12):e01876. doi:10.1002/brb3.1876. 33034127 PMC7749573

[39] Xiao P, Ding S, Duan Y, et al. Effect of light therapy on cancer-related fatigue: a systematic review and meta-analysis. J Pain Symptom Manage. 2022;63(2):e188–e202. doi:10.1016/j.jpainsymman.2021.09.010. 34563631

[40] Ono H, Taguchi T, Kido Y, et al. The usefulness of bright light therapy for patients after oesophagectomy. Intensive Crit Care Nurs. 2011;27(3):158–166. doi:10.1016/j.iccn.2011.03.003. 21511473

[41] Ancoli-Israel S, Martin JL, Kripke DF, et al. Effect of light treatment on sleep and circadian rhythms in demented nursing home patients. J Am Geriatr Soc. 2002;50(2):282–289. doi:10.1046/j.1532-5415.2002.50060.x. 12028210 PMC2764401

[42] Figueiro MG, Hunter CM, Higgins P, et al. Tailored lighting intervention for persons with dementia and caregivers living at home. Sleep Health. 2015;1(4):322–330. doi:10.1016/j.sleh.2015.09.003. 27066526 PMC4822719

[43] Münch M, Schmieder M, Bieler K, et al. Bright light delights: effects of daily light exposure on emotions, rest activity cycles, sleep and melatonin secretion in severely demented patients. Curr Alzheimer Res. 2017;14(10):1063–1075. doi:10.2174/1567205014666170523092858. 28545364

[44] Riemersma-van der Lek RF, Swaab DF, Twisk J, et al. Effect of bright light and melatonin on cognitive and noncognitive function in elderly residents of group care facilities: a randomized controlled trial. JAMA. 2008;299(22):2642–2655. doi:10.1001/jama.299.22.2642. 18544724

[45] Bromundt V, Wirz-Justice A, Boutellier M, et al. Effects of a dawn-dusk simulation on circadian rest-activity cycles, sleep, mood and well-being in dementia patients. Exp Gerontol. 2019;124:110641. doi:10.1016/j.exger.2019.110641. 31252161

[46] Wahnschaffe A, Nowozin C, Haedel S, et al. Implementation of dynamic lighting in a nursing home: impact on agitation but not on rest-activity patterns. Curr Alzheimer Res. 2017;14(10):1076–1083. doi:10.2174/1567205014666170608092411. 28595522

[47] Videnovic A, Klerman EB, Wang W, et al. Timed light therapy for sleep and daytime sleepiness associated with parkinson disease: a randomized clinical trial. JAMA Neurol. 2017;74(4):411–418. doi:10.1001/jamaneurol.2016.5192. 28241159 PMC5470356

[48] Graw P, Recker S, Sand L, et al. Winter and summer outdoor light exposure in women with and without seasonal affective disorder. J Affect Disord. 1999;56(2-3):163–169. doi:10.1016/s0165-0327(99)00037-3. 10701473

[49] Burns AC, Saxena R, Vetter C, et al. Time spent in outdoor light is associated with mood, sleep, and circadian rhythm-related outcomes: a cross-sectional and longitudinal study in over 400,000 UK Biobank participants. J Affect Disord. 2021;295:347–352. doi:10.1016/j.jad.2021.08.056. 34488088 PMC8892387

[50] Dresp-Langley B. Children’s Health in the Digital Age. Int J Environ Res Public Health. 2020;17(9):3240. doi:10.3390/ijerph17093240. 32384728 PMC7246471

[51] Bhandary SK, Dhakal R, Sanghavi V, et al. Ambient light level varies with different locations and environmental conditions: potential to impact myopia. PloS One. 2021;16(7):e0254027. doi:10.1371/journal.pone.0254027. 34234353 PMC8263252

[52] Hébert M, Dumont M, Paquet J. Seasonal and diurnal patterns of human illumination under natural conditions. Chronobiol Int. 1998;15(1):59–70. doi:10.3109/07420529808998670. 9493715

[53] Hartmeyer SL, Andersen M. Towards a framework for light-dosimetry studies: quantification metrics. Light Res Technol. 2024;56(4):337–365. doi:10.1177/14771535231170500

[54] Hartmeyer SL, Webler FS, Andersen M. Towards a framework for light-dosimetry studies: methodological considerations. Light Res Technol. 2022;55(4-5):377–399. doi:10.1177/14771535221103258.

[55] Spitschan M, Smolders K, Vandendriessche B, et al. Verification, analytical validation and clinical validation (V3) of wearable dosimeters and light loggers. Digital Health. 2022;8:205520762211448. 20552076221144858. doi:10.1177/20552076221144858. PMC9806438 36601285

[56] Stampfli JR, Schrader B, Di Battista C, et al. The light-dosimeter: a new device to help advance research on the non-visual responses to light. Light Res Technol. 2023;55(4-5):474–486. doi:10.1177/14771535221147140 PMC10353031 37469656

[57] Mohamed A, Kalavally V, Cain SW, et al. Wearable light spectral sensor optimized for measuring daily α-opic light exposure. Opt Express. 2021;29(17):27612–27627. doi:10.1364/OE.431373. 34615174

[58] CIE S 026/E:2018 CIE system for metrology of optical radiation for iprgc-influenced responses to light. Vienna CIE Central Bureau. Color Res Appl. 2018;44(2):316. doi:10.1002/col.22350.

[59] Spitschan M, Kervezee L, Lok R, et al. ENLIGHT: a consensus checklist for reporting laboratory-based studies on the non-visual effects of light in humans. eBioMedicine. 2023;98:104889. doi:10.1016/j.ebiom.2023.104889. 38043137 PMC10704221

[60] Spitschan M, Stefani O, Blattner P, et al. How to report light exposure in human chronobiology and sleep research experiments. Clocks Sleep. 2019;1(3):280–289. doi:10.3390/clockssleep1030024. 31281903 PMC6609447

[61] Hommes V, Giménez MC. A revision of existing Karolinska Sleepiness Scale responses to light: a melanopic perspective. Chronobiol Int. 2015;32(6):750–756. doi:10.3109/07420528.2015.1043012. 26102373

[62] Brown TM. Melanopic illuminance defines the magnitude of human circadian light responses under a wide range of conditions. J Pineal Res. 2020;69(1):e12655. doi:10.1111/jpi.12655. 32248548

[63] Schöllhorn I, Stefani O, Lucas RJ, et al. Melanopic irradiance defines the impact of evening display light on sleep latency, melatonin and alertness. Commun Biol. 2023;6(1):228. doi:10.1038/s42003-023-04598-4. 36854795 PMC9974389

[64] Cajochen C, Stefani O, Schöllhorn I, et al. Influence of evening light exposure on polysomnographically assessed night-time sleep: a systematic review with meta-analysis. Lighting Research & Technology (London, England: 2001). 2022;54(6):609–624. doi:10.1177/14771535221078765.

[65] Schöllhorn I, Stefani O, Lucas RJ, et al. The impact of pupil constriction on the relationship between melanopic EDI and melatonin suppression in young adult males. J Biol Rhythms. 2024;39(3):282–294. 7487304241226466. doi:10.1177/07487304241226466. 38348477 PMC11141089

[66] Zeeuw J. D, Papakonstantinou A, Nowozin C, et al. Living in biological darkness: objective sleepiness and the pupillary light responses are affected by different metameric lighting conditions during daytime. J Biol Rhythms. 2019;34(4):410–431. doi:10.1177/0748730419847845. 31156018 PMC6637815

[67] Chellappa SL. Individual differences in light sensitivity affect sleep and circadian rhythms. Sleep. 2021;44(2). doi:10.1093/sleep/zsaa214. PMC7879412 33049062

[68] Lucas RJ, Peirson SN, Berson DM, et al. Measuring and using light in the melanopsin age. Trends Neurosci. 2014;37(1):1–9. doi:10.1016/j.tins.2013.10.004. 24287308 PMC4699304

[69] Benedetti M, Maierová L, Cajochen C, et al. Optimized office lighting advances melatonin phase and peripheral heat loss prior bedtime. Sci Rep. 2022;12(1):4267. doi:10.1038/s41598-022-07522-8. 35277539 PMC8917232

[70] He S, Yan Y. Impact of advance light exposure on assembly-line workers’ subjective work alertness and sleep quality. Light Res Technol. 2023;55(2):105–128. doi:10.1177/14771535221078763

[71] Peeters ST, Smolders K, Vogels I, et al. Less is more? Effects of more vs. less electric light on alertness, mood, sleep and appraisals of light in an operational office. J Environ Psychol. 2021;74:101583. doi:10.1016/j.jenvp.2021.101583.

[72] Smolders KC, Peeters HJ, Vogels ST, et al. Investigation of dose-response relationships for effects of white light exposure on correlates of alertness and executive control during regular daytime working hours. J Biol Rhythms. 2018;33(6):649–661. doi:10.1177/0748730418796438. 30198360 PMC6236584

[73] Circadian lighting. Research Insight 01. Chartered Institution of Building Services Engineers. https://www.cibse.org/knowledge-research/knowledge-portal/research-insight-01-circadian-lighting.

[74] Circadian lighting design WELL Standard [accessed 19 July 2024]; 2020. https://standard.wellcertified.com/light/circadian-lighting-design

[75] Beuth Verlag GmbH. DIN SPEC 67600:2013-04, Biologisch wirksame Beleuchtung - Planungsempfehlungen; 2013. doi:10.31030/1941792.

[76] DIN/TS 5031-100:2021-11. Strahlungsphysik im optischen Bereich und Lichttechnik_- Teil_100: Über das Auge vermittelte, melanopische Wirkung des Lichts auf den Menschen_- Größen, Symbole und Wirkungsspektren. Berlin: Beuth Verlag GmbH; 2021.

[77] Cheshmeh Noor M, Revell V, Mehdizadeh Saradj F, et al. The impact of wavelength on acute non-visual responses to light: a systematic review and meta-analysis. Brain Res. 2023;1816:148470. doi:10.1016/j.brainres.2023.148470. 37364848

[78] Mu Y-M, Huang X-D, Zhu S, et al. Alerting effects of light in healthy individuals: a systematic review and meta-analysis. Neural Regen Res. 2022;17(9):1929–1936. doi:10.4103/1673-5374.335141. 35142669 PMC8848614

[79] Pachito DV, Eckeli AL, Desouky AS, et al. Workplace lighting for improving alertness and mood in daytime workers. Cochr Database Syst Rev. 2018;2018(3). doi:10.1002/14651858.CD012243.pub2. PMC6494162 29498416

[80] Nixon A, Robillard R, Leveille C, et al. Assessing the effects of polychromatic light exposure on mood in adults: a systematic review contrasting α-opic equivalent daylight illuminances. LEUKOS. 2023;20(2):127–147. doi:10.1080/15502724.2023.2219017.

[81] Kompier ME, Smolders K, Kort Y. D A systematic literature review on the rationale for and effects of dynamic light scenarios. Build Environ. 2020;186:107326. doi:10.1016/j.buildenv.2020.107326.

[82] Xu Q, Lang CP. Revisiting the alerting effect of light: A systematic review. Sleep Med Rev. 2018;41:39–49. doi:10.1016/j.smrv.2017.12.001. 29398582

[83] Souman JL, Tinga AM, Te Pas SF, et al. Acute alerting effects of light: a systematic literature review. Behav Brain Res. 2018;337:228–239. doi:10.1016/j.bbr.2017.09.016. 28912014

[84] Xiao H, Cai H, Li X. Non-visual effects of indoor light environment on humans: a review. Physiol Behav. 2021;228:113195. doi:10.1016/j.physbeh.2020.113195. 33022281

[85] Prayag AS, Münch M, Aeschbach D, et al. Light modulation of human clocks, wake, and sleep. Clocks Sleep. 2019;1(1):193–208. doi:10.3390/clockssleep1010017. 32342043 PMC7185269

[86] Figueiro MG, Nagare R, Price LL. Non-visual effects of light: how to use light to promote circadian entrainment and elicit alertness. Light Res Technol. 2018;50(1):38–62. doi:10.1177/1477153517721598. 30416392 PMC6221201

[87] Lok R, Smolders KCHJ, Beersma DGM, et al. Light, Alertness, and Alerting Effects of White Light: a Literature Overview. J Biol Rhythms. 2018;33(6):589–601. doi:10.1177/0748730418796443. 30191746 PMC6236641

[88] Stephenson KM, Schroder CM, Bertschy G, et al. Complex interaction of circadian and non-circadian effects of light on mood: shedding new light on an old story. Sleep Med Rev. 2012;16(5):445–454. doi:10.1016/j.smrv.2011.09.002. 22244990

[89] Cajochen C. Alerting effects of light. Sleep Med Rev. 2007;11(6):453–464. doi:10.1016/j.smrv.2007.07.009. 17936041

[90] Boyce P. Human factors in lighting. Boca Raton, FL CRC Press; 2014.

[91] EN 12464-1:2011 - Light and lighting - Lighting of work places - Part 1: Indoor work places. 2022. https://standards.iteh.ai/catalog/standards/cen/75239d59-3e2c-4c3a-b262-e1a80fe62a6e/en-12464-1-2011

[92] Esposito T, Houser K. Correlated color temperature is not a suitable proxy for the biological potency of light. Sci Rep. 2022;12(1):20223. doi:10.1038/s41598-022-21755-7. 36418869 PMC9684473

[93] Vetter C, Phillips AJK, Silva A, et al. Light me up? why, when, and how much light we need. J Biol Rhythms. 2019;34(6):573–575. doi:10.1177/0748730419892111. 31813350

[94] Ali MR. Pattern of EEG recovery under photic stimulation by light of different colors. Electroencephalogr Clin Neurophysiol. 1972;33(3):332–335. doi:10.1016/0013-4694(72)90162-9. 4114919

[95] Blume C, Cajochen C, Schöllhorn I, et al. Effects of calibrated blue–yellow changes in light on the human circadian clock. Nat Hum Behav. 2023;8(3):590–605. doi:10.1038/s41562-023-01791-7. 38135734 PMC10963261

[96] Kelly R. Lighting as an integral part of architecture. College Art Journal. 1952;12(1):24–30. doi:10.2307/773361.

[97] Recommended practice for office lighting. ANSI/IES RP-1-12. Illuminating Society of North America. New York, NY.

[98] Acosta I, León J, Bustamante P. Daylight spectrum index: a new metric to assess the affinity of light sources with daylighting. Energies. 2018;11(10):2545. doi:10.3390/en11102545.

[99] Katia R, Elsayed N, Rakha T. Evaluating Daylighting Performance Metrics in LEED v4 for Commercial Office Buildings: what Criteria is Missing to Enhance the Occupant Visual Performance and Comfort? In Proceedings of Building Simulation 2021: 17th Conference of IBPSA. Building Simulation Conference Proceedings. KU Leuven. 2021. doi:10.26868/25222708.2021.30567.

[100] Adamsson M, Laike T, Morita T. Annual variation in daily light exposure and circadian change of melatonin and cortisol concentrations at a northern latitude with large seasonal differences in photoperiod length. J Physiol Anthropol. 2016;36(1):6. doi:10.1186/s40101-016-0103-9. 27435153 PMC4952149

[101] Kawasaki A, Wisniewski S, Healey B, et al. Impact of long-term daylight deprivation on retinal light sensitivity, circadian rhythms and sleep during the Antarctic winter. Sci Rep. 2018;8(1):16185. doi:10.1038/s41598-018-33450-7. 30385850 PMC6212492

[102] Owen J, Arendt J. Melatonin suppression in human subjects by bright and dim light in antarctica: time and season-dependent effects. Neurosci Lett. 1992;137(2):181–184. doi:10.1016/0304-3940(92)90399-r. 1584458

[103] Stothard ER, McHill AW, Depner CM, et al. Circadian entrainment to the natural light-dark cycle across seasons and the weekend. Curr Biol. 2017;27(4):508–513. doi:10.1016/j.cub.2016.12.041. 28162893 PMC5335920

[104] Wehr TA. Melatonin and seasonal rhythms. J Biol Rhythms. 1997;12(6):518–527. doi:10.1177/074873049701200605. 9406025

[105] Wright KP, McHill AW, Birks BR, et al. Entrainment of the human circadian clock to the natural light-dark cycle. Curr Biol. 2013;23(16):1554–1558. doi:10.1016/j.cub.2013.06.039. 23910656 PMC4020279

[106] Hébert M, Martin SK, Lee C, et al. The effects of prior light history on the suppression of melatonin by light in humans. J Pineal Res. 2002;33(4):198–203. doi:10.1034/j.1600-079x.2002.01885.x. 12390501 PMC3925650

[107] Schöllhorn I, Stefani O, Blume C, et al. Seasonal variation in the responsiveness of the melanopsin system to evening light: why we should report season when collecting data in human sleep and circadian studies. Clocks Sleep. 2023;5(4):651–666. doi:10.3390/clockssleep5040044. 37987395 PMC10660855

[108] Smith KA, Schoen MW, Czeisler CA. Adaptation of human pineal melatonin suppression by recent photic history. J Clin Endocrinol Metab. 2004;89(7):3610–3614. doi:10.1210/jc.2003-032100. 15240654

[109] Chang A-M, Scheer FAJL, Czeisler CA, et al. Direct effects of light on alertness, vigilance, and the waking electroencephalogram in humans depend on prior light history. Sleep. 2013;36(8):1239–1246. doi:10.5665/sleep.2894. 23904684 PMC3700721

[110] Münch M, Nowozin C, Regente J, et al. Blue-enriched morning light as a countermeasure to light at the wrong time: effects on cognition, sleepiness, sleep, and circadian phase. Neuropsychobiology. 2016;74(4):207–218. doi:10.1159/000477093. 28637029

[111] Wienold J. Dynamic daylight glare evaluation. Eleventh International IBPSA Conference, Glasgow; 2009. pp. 944–951.

[112] Troland LT. The theory and practice of the artificial pupil. Psychological Review. 1915;22(3):167–176. doi:10.1037/h0073849.

[113] Eto T, Ohashi M, Nagata K, et al. Crystalline lens transmittance spectra and pupil sizes as factors affecting light-induced melatonin suppression in children and adults. Ophthalmic Physiol Opt. 2021;41(4):900–910. doi:10.1111/opo.12809. 33772847

[114] Zauner J, Broszio K, Bieske K. Influence of the human field of view on visual and non-visual quantities in indoor environments. 2023. 10.3390/clockssleep5030032 PMC10530223 37754350

[115] Council of the European Union. Council Directive 92/75/EEC of 22 September 1992 on the indication by labelling and standard product information of the consumption of energy and other resources by household appliances. 31992L0075. 1992.

[116] LEED rating system U.S. Green Building Council; 2024. https://www.usgbc.org/leed.

[117] SNBS Hochbau. Standard Nachhaltiges Bauen Schweiz. SNBS. 2023.

[118] FAIR Principles. GO FAIR; 2024. https://www.go-fair.or/fair-principles.

[119] Kronauer RE, Forger DB, Jewett ME. Quantifying human circadian pacemaker response to brief, extended, and repeated light stimuli over the phototopic range. J Biol Rhythms. 1999;14(6):500–515. doi:10.1177/074873049901400609. 10643747

[120] Murray JM, Magee M, Sletten TL, et al. Light-based methods for predicting circadian phase in delayed sleep–wake phase disorder. Sci Rep. 2021;11(1):10878. doi:10.1038/s41598-021-89924-8. 34035333 PMC8149449

[121] Skeldon AC, Dijk D-J, Meyer N, et al. Extracting circadian and sleep parameters from longitudinal data in schizophrenia for the design of pragmatic light interventions. Schizophr Bull. 2022;48(2):447–456. doi:10.1093/schbul/sbab124. 34757401 PMC8886588

[122] Tekieh T, Lockley SW, Robinson PA, et al. Modeling melanopsin-mediated effects of light on circadian phase, melatonin suppression, and subjective sleepiness. J Pineal Res. 2020;69(3):e12681. doi:10.1111/jpi.12681. 32640090

