# Supplementary material for: Towards an evidence-based integrative lighting score: a proposed multi-level approach
Source: Ann Med. 2024 Jul 25;56(1):2381220. doi: 10.1080/07853890.2024.2381220 (PMC11275531; doi:10.1080/07853890.2024.2381220)
Supplement: Commentary_Supplement_EBLS_Stefani_et_al_final_V2.docx [file IANN_A_2381220_SM3216.docx]

Towards an evidence-based integrative lighting score:

a proposed multi-level approach

Supplement

Authors: Oliver Stefani^1^ (PhD), Isabel Schöllhorn^2^ (MSc), Mirjam Münch^2^ (PhD)

^1^ Lucerne University of Applied Sciences and Arts, Lucerne School of Engineering and Architecture, 6048 Horw, Switzerland; ^2^Centre for Chronobiology, Psychiatric Hospital of the University of Basel, Research Cluster Molecular Cognitive Neuroscience, University of Basel, 4002 Basel, Switzerland

To identify gaps in current knowledge of light effects during the day, we used recently published systematic literature reviews and/or meta-analyses. Based on three categories with their characteristics (magnitude, distribution, spectral tuning) and the three selected effects of neurobehavioral actions in humans (mood, alertness and cognition), a mini-review on non-visual light effects was conducted in accordance with PRISMA criteria (Preferred Reporting Items for Systematic reviews and Meta-Analyses). Eligibility criteria were that (1) the studies included healthy humans, (2) alertness, mood or cognitive performance was assessed, (3) illuminance levels, direction of measurement and duration of light exposure were reported, (4) the type of light source and/or spectral characteristics and/or photon density were reported, and (5) the active light and control light exposure occurred during the daytime between 6:00 a.m. to 9:00 p.m. (or no later than 3 hours before bedtime) and lasted for at least 30 minutes. There were no restrictions regarding study design, prior light history, sex, or age of the participants. Searches were performed in the PubMed® and Web of Science databases on 23 June 2023.

The search string included the following items: ((Light [Title]) OR (Lighting [Title]) OR (color [Title]) OR (brightness [Title]) OR (Correlated colour temperature [Title]) OR (Melanopic [Title]) OR (Spectrum [Title]) OR (Twilight [Title]) OR (Wavelength [Title]) OR (Spectral composition [Title]) OR (Metameric [Title]) OR (Metamerism [Title]) OR (Silent substitution [Title]) OR (Colour signal [Title]) OR (Photon Flux [Title]) OR (Illuminance [Title]) OR (Irradiance [Title]) OR (Luminance [Title]) OR (Radiance [Title]) OR (Lux [Title]) OR (Candela [Title]) OR (Ultradian [Title]) OR (Luminaire [Title]) OR (Retinal area [Title]) OR (Solid angle [Title])) AND ((comfort [Title]) OR (well-being [Title]) OR (mood [Title]) OR (performance [Title]) OR (changing [Title]) OR (Alertness [Title]) OR (alerting [Title])) NOT ((glasses [Title]) OR (disorders [Title]) OR (drug [Title]) OR (bipolar [Title]) OR (autism [Title])). Of the 1165 studies identified in PubMed during the initial stage of the literature search, 198 full-text articles were retrieved and assessed according to the selection criteria.

The initial Web of Science search identified 834 studies. After removing duplicates (1071), the search yielded 928 articles, that were screened by O.S. against the selection criteria. A total of 172 remaining studies were assessed for eligibility. O.S. screened titles, abstracts and full texts, in this order, for inclusion/exclusion. Based on our search strategy, we first identified all papers that are relevant to these criteria and included daytime lighting. For this manuscript, we focused on published literature reviews and meta-analyses . From the total list of references, we identified 19 literature reviews, recommendations and meta-analysis on which we base our conclusions in this commentary.

Supplemental Figure 1: Flow – chart for the search process and numbers of included and excluded papers.

**Identification of studies via databases and registers**

Records removed *before screening*:

Duplicate records removed (n = 1071)

Records marked as ineligible by automation tools (n = 0)

Records removed for other reasons (n = 0)

Records identified from:

Databases (n = 2)

Registers (n = 1999)

**Identification**

Records screened

(n = 928)

Records excluded

(n = 756)

Reports sought for retrieval

(n = 172)

Reports not retrieved

(n = 0)

**Screening**

Reports assessed for eligibility

(n = 172)

Reports excluded:

night time studies (n = 61)

animal studies (n = 18)

not reviews, recommendations or meta-analysis (n = 74)

Studies included in review

(n = 19)

Reports of included studies

(n = 19)

**Included**
